# Hints for the Sick-Room

**Published:** 1888-09-22

**Authors:** M. M. Basil

**Affiliations:** Author of "The Commoner Accidents to Life and Limb."


### HINTS FOR THE SICK-ROOM.

BY M. M. BASIL, M.A., M.B., C.M., Author of "The Commoner Accidents to Life and Limb."

ADMINISTRATION OF MEDICINES (*continued*).

GREAT harm may be done to patients if the end of the catheter is first embedded in the irritating and often infectious discharges, and then carried onwards into the urethra or bladder. Not a few of the extremely troublesome cases of chronic inflammation of the bladder are forms of micro-organic infection. The exact proportion of cases in these circumstances, where the catheter has been the means of carrying infective pus from the external to the internal parts has yet to be determined, but the fact that the instrument has been the means of this extension of disease from one area to another is beyond dispute.

It will be seen that the view taken of the subject here differs materially from that found in many of the text-books on nursing. The rules found in these text-books have been borrowed largely from manuals on the minor surgical operations, most of which are the productions of general surgeons not especially acquainted with the questions involved in the catheterisation of the female bladder. It has therefore come about that many rules laid down on a subject by men professedly unacquainted with all its bearings have come to be looked upon as orthodox merely by dint of repetition by generations of successive authors on minor surgery and nursing. It would be invidious to mention any one book where so many are to blame; it would seem, however, that according to most of them the accomplished and technical guiding in the dark of a catheter into a woman's bladder constitutes the height of artistic and "surgical" nursing. The orthodox exactitude with which this scientific flourishing at random of surgical weapons must be accomplished is insisted upon everywhere with sufficient emphasis, both borrowed and original. Believing that the risks of artificial inoculation by the catheter are not imaginary, I feel no hesitation in asserting that many of the usual rules for the introduction of the catheter ought to be more honoured in the breach that the observance.

After pelvic operations the passage under the clothes of the catheter is not attended with similar risks, because no surgeon would, from choice, subject any patient to such an operation until all local diseases accompanied with irritating discharges are removed.

It is best to pass the catheter on tried cases first, that is, on in-patients who have had the instrument introduced previously without any bad results. Sometimes extreme irritation follows the operation, even in experienced hands. You should therefore be in no hurry to undertake its introduction for the first time in any patient. Usually, however, the so-called spasms of the urethra, irritable urethra, and other euphonisms, are well understood to be indirect modes of speech for concealing ideas which one finds inconvenient to put into clear expression. Some of the cases of prolonged irritability of the urethra following first catheterisation are really forms of urethral ulcer, very much resembling anal ulcers, and capable of rapid cure by a similar treatment, viz., complete division of the base of the ulcer by over-distension of the urethra.

It is easier to pass the instrument in thin than in stout people. You should never catheterise unmarried patients without special instruction to do so, and never subject any patient to the procedure unless there are reasons compelling you to that course.

Before withdrawing the catheter, the finger must be placed against its open end, or the indiarubber tube must be gripped, so as to prevent the soiling of the sheets by the fluid which is in the tube. A similar precaution is necessary during the introduction of the instrument.

Tapping or squeezing an over-distended bladder is a procedure not by any means free from danger, as it may rupture the organ. You should therefore be cautious how you move the patient about, or how you touch the parts over the bladder in such circumstances.

The different positions in which patients, and especially female patients, are placed for investigation of disease and operative interference have been referred to more than once, and must be very briefly described. The positions which are in most frequent use will be alluded to, because these alone fall under the entire charge of the nurse. Complicated postures necessary for very rare operations will be left out of consideration, as the surgeon himself will direct the assistants in such circumstances.

The position expressed simply by the term on the back is plain enough; but it is necessary to remember that the shoulders must be supported moderately, and the thighs flexed so as to relax the abdominal walls. If a pelvic examination is to be made in this position, the knees must be bent and drawn upwards, so that the limbs rest on the soles of the feet, while the heels are kept apart at a distance of four to eight inches; the knees must not be separated from each other so widely as the heels.

The lithotomy position resembles the last one very much, but the pelvis is drawn past the edge of the bed and often raised considerably. Each leg is bent on the thigh and supported by a nurse. In prolonged operations this is best done by standing well forward, and then leaning back on the limb placed between and behind the armpit of the nurse. The limb is thus retained in position by the weight of the attendant's body, and not by any especial muscular effort. It is often useful to keep the patient's feet apart at a fixed distance by means of the crutch.

The side positions consist either in placing the patient simply on one or other side, with the knees bent and drawn up, or in placing the patient mainly on the left side, but arranging the body in such a manner as to have her lying partially on her face and thorax. The pelvis here becomes the highest point of the body. This latter modification of side positions is known as Sims' position, and must become thoroughly familiar to every nurse. Several essential conditions must be fulfilled for placing a patient in this position. The abdomen and thorax must be quite free from any restraint by clothing; the left arm and shoulder must be drawn away from under the patient, so that she lies on the thorax and not on the arm or shoulder; the right shoulder rotated completely towards the bed, so that it nearly touches the bed; the spinal column and body must not be bent; the head may be slightly flexed, but it is best to keep it in the same line with the spine; the trunk must be placed neither across the bed nor along it, but in a diagonal position, with an inclination rather to the transverse than the longitudinal axis of the bed; the thighs must be flexed, the right, or upper one, a little more than the lower, and the heels drawn upwards, and away from the corner occupied by the surgeon. It is best to request the patient to lie flat on the back, and then to place her in the position required. Mere verbal directions to her will only confuse her and add to the difficulties. A good deal of the success in securing this position will depend upon the rotation which is given to the pelvis, or lower portion of the trunk, while drawing it past the edge of the bed. The pelvis should be drawn out with a rotary movement—that is, its lower or left portion should be drawn towards the nurse and the foot of the bed, while the upper portion is rotated away from the nurse and towards the head of the bed.

